# Rickettsioses Seropositivity in Malaysia: A Six-Year Trend, 2016–2021

**DOI:** 10.3390/tropicalmed10080205

**Published:** 2025-07-24

**Authors:** Bee Yong Tay, Fashihah Sherina Abdul Hadi Sabri, Zamtira Seman, Norlela Othman, Haida Subakir, Zahrul Laili Abd Hadi, Adilahtul Bushro Zaini, Norli Anida Abdullah, Nur Anisah Mohamed, Mohammad Yazid Abdad, Siti Roszilawati Ramli

**Affiliations:** 1 Bacteriology Unit, Infectious Disease Research Centre, Institute for Medical Research, National Institutes of Health, Ministry of Health, Shah Alam 40170, Selangor, Malaysia; 2 Sector for Biostatistics & Data Repository, National Institutes of Health, Ministry of Health, Shah Alam 40170, Selangor, Malaysia; 3 Pathology Department, Hospital Sultanah Nur Zahirah, Kuala Terengganu 20400, Terengganu, Malaysia; 4 Pathology Department, Hospital Sultan Ismail, Johor Bahru 81100, Johor, Malaysia; 5 Pathology Department, Hospital Raja Permaisuri Bainun, Ipoh 30450, Perak, Malaysia; 6 Pathology Department, Hospital Sungai Buloh, Sungai Buloh 47000, Selangor, Malaysia; 7 Mathematics Division, Centre for Foundation Studies in Science, Universiti Malaya, Kuala Lumpur 50603, Selangor, Malaysia; 8 Center for Data Analytics Consultancy and Services, Faculty of Science, Universiti Malaya, Kuala Lumpur 50603, Selangor, Malaysia; 9 Institute of Mathematical Sciences, Faculty of Science, Universiti Malaya, Kuala Lumpur 50603, Selangor, Malaysia; 10 Mahidol Oxford Tropical Medicine Research Unit, Faculty of Tropical Medicine, Mahidol University, Bangkok 10400, Thailand; 11 Centre for Tropical Medicine, Nuffield Department of Clinical Medicine, John Radcliffe Hospital, Oxford OX3 7BN, UK

**Keywords:** rickettsioses, seropositivity, Malaysia, scrub typhus (ST), typhus group rickettsioses (TGR), spotted fever group rickettsioses (SFGR)

## Abstract

Background: Rickettsioses are diseases caused by obligate intracellular non-motile coccobacilli transmitted via arthropods. The most common rickettsioses are scrub typhus (ST), typhus group rickettsioses (TGR), and spotted fever group rickettsioses (SFGR). This study aims to provide information and insight into rickettsioses seropositivity among suspected patients in East and Peninsular Malaysia over a six-year period from 2016 to 2021. Methodology/Principal Findings: Data obtained from four state hospitals and one national research institute providing rickettsial serological testing were analyzed using the IBM SPSS (Statistical Package for the Social Sciences) software program. The six-year analysis revealed that ST had the highest number of seropositivity cases, followed by TGR, and SFGR, for both immunoglobulin M (IgM) and immunoglobulin G (IgG) antibodies. Of the 3228 samples, 21.6%, 16.1%, and 13.9% of suspected patients were IgM seropositive for ST, TGR, and SFGR, respectively. IgG seropositivity for ST was 21.9%, followed by TGR at 21.4%, and SFGR at 17.2% among suspected rickettsioses cases. All regions in Malaysia were significantly associated with IgM seropositivity for ST, TGR, and SFGR. IgM seropositivity for SFGR was significantly higher in females. Age group 41–65 years was highly associated with IgG seropositivity for ST, TGR, and SFGR. Conclusions/Significance: Analysis of six-year data on ST, TGR, and SFGR seropositivity in Malaysia revealed variations across regions, age groups, and genders. This seropositivity study underscores ST, TGR, and SFGR as possible causes of acute febrile illness among patients suspected of rickettsial disease in Malaysia. The findings contributed to the awareness of reemerging rickettsioses and warrant public health interventions that may reduce the incidence of rickettsioses in Malaysia. Abstract summary: Scrub typhus (ST), typhus group rickettsioses (TGR), and spotted fever group rickettsioses (SFGR) are significant global public health concerns. Our results showed that the highest number of IgM and IgG seropositivity cases was observed for ST, followed by TGR and SFGR. All regions in Malaysia were significantly associated with IgM seropositivity for ST, TGR, and SFGR. East Malaysia exhibited significantly higher seropositivity for ST, TGR, and SFGR than other regions in Malaysia. IgM seropositivity for SFGR was significantly higher in females. The age group 41–65 years was highly associated with IgG seropositivity for ST, TGR, and SFGR. This study highlights the value of serological data in uncovering the hidden burden of disease in Malaysia. In addition, the findings contributed to bridging knowledge gaps on the limited data from Malaysia spanning extended periods, despite being one of the countries in the endemic Tsutsugamushi Triangle. The findings from this study may direct future research on rickettsioses and warrant public health interventions in Malaysia.

## 1. Introduction

Rickettsioses are zoonotic infections caused by obligate intracellular bacteria grouped in the family Rickettsiaceae of α-proteobacteria. The causative organisms of rickettsioses comprise two genera, *Orientia* and *Rickettsia*. *Orientia tsutsugamushi* causes scrub typhus (ST), also known as tsutsugamushi disease, and is transmitted by chigger mites. Two additional *Orientia* species were discovered, increasing the membership to three. *Orientia chuto* was isolated from a patient in Dubai [1], and *Candidatus Orientia chiloensis* was found in Chile [2]. The diversity of *Rickettsia* species can be classified into five groups—(i) the Bellii Group, which consists of *Rickettsia bellii*, (ii) the recently proposed transitional group, which includes *Rickettsia akari* (mite-borne), *Rickettsia australis* (tick-borne), and *Rickettsia felis* (flea-borne), (iii) the typhus group (TG), which causes typhus group rickettsioses (TGR), consists of two members of the *Rickettsia* genus - *Rickettsia typhi*, an agent of murine typhus (also known as endemic typhus), and *Rickettsia prowazekii*, which causes epidemic typhus, (iv) the Tamurae-Ixodes Group (TIG) which consists of *Rickettsia tamurae* (tick-borne), and (v) the spotted fever group (SFG) *Rickettsia*, which contains many species. This group is mainly transmitted from ticks, mites, or lice to mammals. Some *Rickettsia* species within the SFG group cause spotted fever group rickettsioses (SFGR) in humans. The list of *Rickettsia* species in the SFG is too numerous to list in the text; thus, we have included an appropriate citation to a recent review that has the full comprehensive list of rickettsial organisms that fall within this classification [3,4,5]. Rickettsioses are largely considered neglected tropical diseases and are major contributors to acute febrile illness in Southeast Asia [6]. These obligate intracellular Gram-negative bacteria are mainly transmitted through the bite of an arthropod vector, such as ticks, fleas, and mites [7]. The main clinical symptoms are fever, rash, lymphadenopathy, and eschar; however, not all patients diagnosed with rickettsioses will have all the clinical findings described [8].

Rickettsial organisms have been found on every continent, with recently reported new *Orientia* and *Orientia*-like organisms [9]. Among all recognized rickettsioses, a greater proportion of the worldwide population is at risk of ST than for any other rickettsial disease. It is estimated that more than one million cases occur annually, with farmers at higher risk [10]. Rickettsioses have been reported in Malaysia for a long time [11,12,13]. Since the 1990s, rickettsial serology has been conducted in tertiary hospitals in Malaysia [14]. However, routine testing for rickettsioses is not commonly performed, despite it being a notifiable disease under the guidelines of the Ministry of Health (MOH) Malaysia [15]. Paired acute-phase and convalescent-phase serum samples are rarely available, which may contribute to the low number of rickettsial infections reported nationwide—only 631 between 1996 and 2015 [16,17]. The rickettsial diseases detected in our setting were ST, typhus group rickettsioses (TGR), and spotted fever group rickettsioses (SFGR). The majority of positive cases were ST infections.

Serological testing played a significant role in the diagnosis of rickettsioses. These tests detected specific antibodies against *O. tsutsugamushi* and *Rickettsia* spp. in the serum of patients and helped confirm recent or past infections. The interpretation of serological results requires consideration of antibody titers and the patient’s clinical context, contributing to the overall diagnosis and management of rickettsial diseases. Methods used for serological testing, including in-house and commercial tests, include indirect immunoperoxidase (IIP) (most common in Asia), immunofluorescence assay (IFA), and enzyme-linked immunosorbent assay (ELISA). The IFA is considered the reference serological method [18,19] and demonstrates a sensitivity of 83–100% and specificity of 99–100% [18]. While specific performance data for the IIP assay are limited, it employs a similar antigen–antibody detection principle as IFA and allows interpretation via light microscopy, making it more accessible for laboratories without fluorescence capabilities [18]. ELISA exhibits more variability depending on assay type, with reported sensitivity between 83% and 100% and specificity between 87% and 100% [18].

Previous studies in Malaysia, based on serological data from eight tertiary hospitals, reflected the endemicity of rickettsial diseases. IgG and/or IgM antibodies to *O. tsutsugamushi*, *R. typhi*, and SFG rickettsiae (TT118) by the IIP test revealed 4.9%, 3.1%, and 2.6% (antibody titers ≥ 400), respectively [14]. Despite the increasing recognition of rickettsioses as a reemerging but neglected zoonotic acute febrile illness, there is a scarcity of current data on rickettsial seropositivity in Malaysia, with limited studies spanning extended periods. Here, we report the seropositivity for ST, TGR, and SFGR in Malaysia from 2016 to 2021. The findings of this study will enhance the understanding and provide an updated overview of rickettsial seropositivity across regions in Malaysia. The data will also contribute to the input from the Southeast Asia region and be made accessible for comparison to a wider geographical area. Furthermore, these data will enhance awareness and assist in public health interventions in the prevention, treatment, control, and policy of these neglected tropical diseases.

## 2. Materials and Methods

### 2.1. Sample Collection

**Figure 1:**
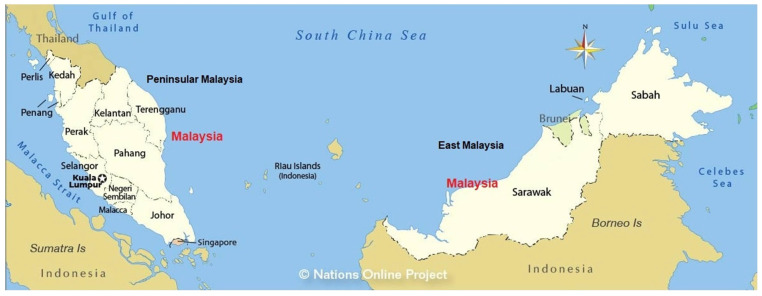
Map of Malaysia showing the five study regions included in rickettsial seropositivity analysis, illustrating the national distribution of cases (modified from: https://www.nationsonline.org/oneworld/map/malaysia_map.htm#google_vignette) (accessed on 4 March 2025) [20]. Peninsular Malaysia is divided into four regions as follows: The northern region includes the states of Perlis, Kedah, and Penang; the central region consists of the federal territory of Kuala Lumpur and the state of Selangor; the East Coast region consists of the states of Kelantan, Terengganu, and Pahang; and the southern region includes the states of Johor, Melaka (Malacca), and Negeri Sembilan. Sabah and Sarawak are part of the East Malaysia region.

A total of 3228 sera of patients suspected of rickettsial diseases from five health centers, consisting four state hospitals and one research institute, were obtained over the 6-year period between 2016 and 2021. Rickettsial diseases were suspected when patients presented with an acute onset of fever with any of these associated clinical findings, such as headache, rash, profuse sweating, myalgia, and gastrointestinal symptoms, eschar, lymphadenopathy, and maculopapular rash (MOH, 2017) [15]. The final sample size of 3228 represents the number of cases included after data cleaning, including the removal of incomplete records and outliers. As this study relied on available laboratory diagnostic submissions across the country rather than prospective recruitment, no formal sample size calculation was performed. The collected samples were compatible with specific clinical guidelines by the MOH, Malaysia, which were followed across centers [15]. All samples with a titer greater than or equal to 1:50 for ST, TGR, and SFGR were requested for convalescence samples to look for a four-fold rise in titer. The four state hospitals in Peninsular Malaysia involved and their respective catchment areas were (i) Hospital Sungai Buloh in Selangor, covering the central region (Federal territories of Kuala Lumpur and the state of Selangor), (ii) Hospital Sultanah Nur Zahirah in Terengganu, covering the East Coast region (the states of Kelantan, Pahang, and Terengganu), (iii) Hospital Raja Permaisuri Bainun in Perak, covering the northern region (the states of Kedah, Penang, Perak, and Perlis), and (iv) Hospital Sultan Ismail in Johor, covering the southern region (the states of Johor, Melaka, and Negeri Sembilan). The Institute for Medical Research reported on the East Malaysia region (the state of Sarawak, located on the northwest coast of Borneo, and Sabah, located in the northeast part of the island of Borneo) (Figure 1). A cross-sectional study was used as the research design. Since our analyses were based on clinically suspected patients, the seropositivity figures should be interpreted with caution and may not represent the wider population.

### 2.2. Sample Testing

Sera collected from suspected patients were tested with rickettsial serology by determining immunoglobulin M (IgM) and immunoglobulin G (IgG) antibodies using the indirect immunoperoxidase procedure described previously [14]. *O. tsutsugamushi* (a mixture of Karp, Kato, and Gilliam serotypes), *R. typhi* Wilmington strain, and *Rickettsia honei* strain TT118 antigens were used as targets for ST, TGR, and SFGR, respectively. Five µL of each serum sample were tested. Serum samples with an anti-rickettsia antibody titer of ≥1:50 were considered seropositive.

### 2.3. Statistical Analysis

Data were analyzed using IBM SPSS (Statistical Package for the Social Sciences) software program, version 28.0.0.0 (SPSS Inc., Chicago, IL, USA). Descriptive statistics were used to summarize the seropositivity of ST, TGR, and SFGR across different regions, age groups, and gender. The age groups (<15, 15–40, 41–65, and >65 years) were chosen to reflect meaningful life stages, including school-age children, early working adults, late working age, and the elderly/retirees. These groups were selected based on their relevance to potential exposure risks and behavioral differences that may affect disease susceptibility and healthcare-seeking behavior. Univariate analysis using binary logistic regression was conducted to identify potential associations. Variables with a *p*-value of less than 0.25 in the univariate analysis were included in the initial multivariate logistic regression model. The final model was developed using the enter method, ensuring that all selected variables were analyzed simultaneously. Multicollinearity among independent variables was assessed, and interaction terms were examined to account for potential effect modifications. The goodness-of-fit of the final model was evaluated using the Hosmer–Lemeshow test, and its predictive accuracy was assessed using a classification table. The strength of the association between risk factors and seropositivity was presented as crude and adjusted odds ratios (AORs) with corresponding 95% confidence intervals. Statistical significance was determined at a *p*-value threshold of <0.05. The Supplementary Materials of this study can be accessed at the Supplementary S1 Document Dataset.

### 2.4. Ethics Statement

This study was registered with the National Medical Research Register (NMRR) and ethically approved by the Medical Research and Ethics Committee (MREC), Ministry of Health, Malaysia, on 24 May 2023, with reference number NMRR-23-01107-JVW. All samples included in this study were post-diagnostic specimens collected as part of routine clinical care and processed according to standard protocols. All samples were de-identified and anonymized prior to study inclusion to ensure patient confidentiality. The requirement for informed consent was waived.

## 3. Results

Between 2016 and 2021, 3228 sera of suspected patients from five health centers were obtained. IgM seropositivity for ST was 21.6%, followed by TGR at 16.1% and SFGR at 13.9%. IgG seropositivity for ST was 21.9%, which was comparable to IgM seropositivity. Meanwhile, seropositivity for TGR was 21.4%, and seropositivity for SFGR was 17.2%, slightly higher than that for IgM. Table 1 presents the demographic characteristics of 3228 patients suspected of rickettsial diseases who were tested for ST, TGR, and SFGR. 

**Figure 2:**
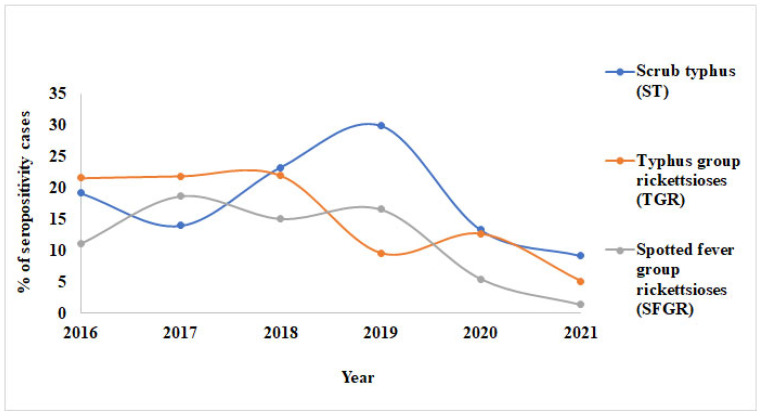
Annual trend of IgM seropositivity (2016–2021), showing fluctuations in acute rickettsial infections over time.

**Figure 3:**
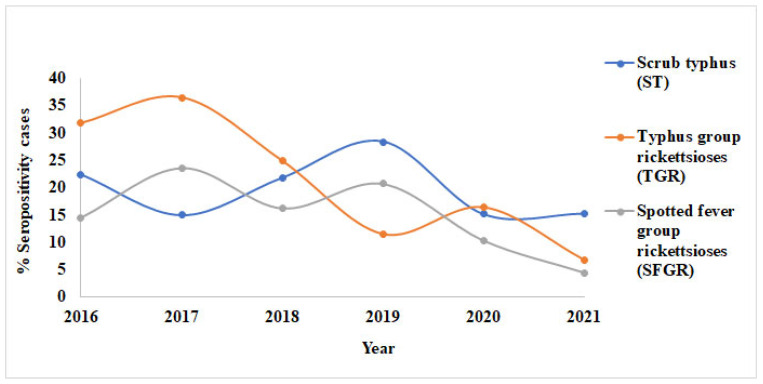
Annual trend of IgG seropositivity (2016–2021), indicating patterns of past exposure across the study period.

IgM and IgG seropositivity for ST decreased in 2017, then increased in 2018 and 2019 before dropping the following year (Figure 2 and Figure 3; Table 2 and Table 3). For IgM, seropositivity for ST decreased between 2020 and 2021; however, IgG showed an almost similar trend. IgM and IgG seropositivity for TGR showed a nearly identical trend from 2016 to 2017 and between 2019 and 2021. The difference between them was shown in 2018, in which the seropositivity for TGR (IgM) increased from the previous year, whereas that for IgG, dropped. Seropositivity for SFGR showed a similar trend for IgM and IgG.

Our data revealed a significant association between IgM seropositivity for ST, TGR, and SFGR and geographic region, with seropositivity observed across all regions of Malaysia. However, Sarawak in the East Malaysia region showed higher association with IgM seropositivity for ST (AOR = 11.24; 95% CI: 5.56, 22.71; *p* < 0.001) (Table 4), TGR (AOR = 4.72; 95% CI: 2.61, 8.52; *p* < 0.001) (Table 5), and SFGR (AOR = 22.53; 95% CI: 7.03, 72.25; *p* < 0.001) (Table 6). Analysis showed that the association between IgM seropositivity for SFGR and genders was significantly lower for males, with AOR = 0.78; 95% CI: 0.63–0.96; *p* = 0.019. Analysis showed that the association between IgM seropositivity for SFGR and genders was significantly lower for males, with AOR = 0.78; 95% CI: 0.63–0.96; *p* = 0.019. There was no significant difference between genders in IgM seropositivity for ST and TGR. The age group > 65 years was significant for TGR (Table 5). The age group >65 years demonstrated significantly lower association with IgM seropositivity for SFGR compared to younger age groups (Table 6).

For IgG antibodies, Sarawak showed the highest ST seropositivity (AOR = 16.19, 95% CI = 7.39, 35.47, *p*-value < 0.001) (Table 7) and SFGR (AOR = 10.87, 95% CI = 5.38, 21.97, *p*-value < 0.001) (Table 8). Data showed that all regions were significantly exposed to ST, TGR, and SFGR except the East Coast region, which was insignificant to TGR (Table 9) and SFGR (Table 8). Sabah has the highest TGR seropositivity (AOR = 5.30, 95% CI = 3.07, 9.14), *p*-value < 0.001) (Table 9). Significant IgG seropositivity for ST, TGR, and SFGR was found across all regions (Table 7, Table 8 and Table 9), except in the East Coast region, where seropositivity for TGR and SFGR was not significant (Table 8 and Table 9), respectively. Sarawak showed significantly higher ST seropositivity (AOR = 16.19; 95% CI: 7.39, 35.47; *p* < 0.001) (Table 7) and SFGR seropositivity (AOR = 10.87; 95% CI: 5.38, 21.97; *p* < 0.001) (Table 8) compared to Southern region. Sabah had higher TGR seropositivity (AOR = 5.30; 95% CI: 3.07, 9.14); *p* < 0.001) (Table 9) compared to Southern region. There was no association between IgG seropositivity and genders for ST and TGR. However, it was significant in SFGR seropositivity where males predominated (AOR = 1.36; 95% CI: 1.11, 1.66; *p* = 0.003) (Table 8). The age group 41–65, was significant in IgG seropositivity for all rickettsioses with ST (AOR = 1.39; 95% Cl: 1.02, 1.91; *p* = 0.039) (Table 7), TGR (AOR = 1.62; 95% Cl: 1.16, 2.27; *p* = 0.005) (Table 9) and SFGR (AOR = 1.75; 95% Cl: 1.21, 2.53; *p* = 0.003) (Table 8). Seropositivity for SFGR also showed significance for the age group >65 years (AOR = 1.77; 95% CI: 1.12, 2.80; *p* = 0.014) (Table 8).

### Rainfall Correlation

**Figure 4:**
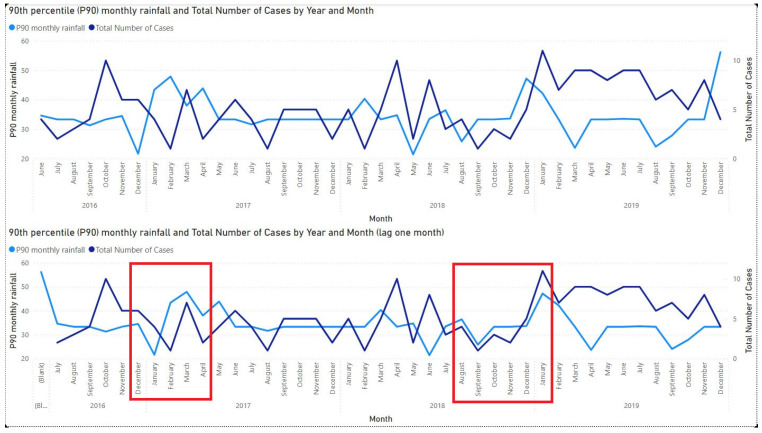
90th percentile (P90) of monthly rainfall and total number of rickettsioses cases from June 2016 to December 2019, shown for zero-month lag (**top panel**) and one-month lag (**bottom panel**) The red boxes indicate a one-month lag pattern relative to rainfall, particularly between December 2016 and April 2017, and August 2018 and January 2019.

The correlation of rainfall with rickettsioses was studied by looking at cases in Sarawak. The 90th percentile is used to represent the monthly rainfall amount to match the monthly data of rickettsioses cases. Figure 4 presents the time series of the 90th percentile of rainfall and the number of rickettsioses cases from June 2016 to December 2019 for the zero-month lag (top panel) and one-month lag (bottom panel). The one-month lag time series plot shows that the number of cases in Sarawak exhibits a clear one-month lag pattern relative to rainfall, particularly between December 2016 and April 2017 as well as August 2018 and January 2019, as indicated by the red boxes. The rainy season in Sarawak usually occurs between September and March (Saadi et al., 2023; Ling et al., 2017) [21,22]. The supplementary materials of this study can be accessed at the Supplementary S2 Document Dataset.

1. Saadi [21]. https://www.sciencedirect.com/science/article/pii/S2212094723000075 (accessed on 2 July 2025).
2. Ling et al. [22]. https://www.pjoes.com/Influence-of-Rainfall-on-the-Physicochemical-Characteristics-of-a-Tropical-River,69439,0,2.html (accessed on 2 July 2025).

## 4. Discussion

The trend of samples collected throughout the year has increased since 2016, as shown in Table 1. It peaked in 2019, then showed a downtrend, and then slowly rose again in 2021. Since the COVID-19 pandemic caused by the SARS-CoV-2 virus in 2020, there has been a significant drop in rickettsioses testing. The Movement Control Order (MCO) enforced nationwide by the Malaysian government between March and May 2020 [23,24,25] to curb the spread of SARS-CoV-2 may have influenced healthcare access to patients who may be suspected of having rickettsial infections. As the nation’s healthcare workforce geared towards COVID-19 testing, laboratory tests such as the IIP may have been reduced for a brief period, resulting in a marked decline in samples during 2020.

Our data revealed that the East Malaysia region was highly associated with IgM and IgG seropositivity for ST, TGR, and SFGR than other regions. The disparity between the two regions in Malaysia may be linked to ecological and entomological factors. Sabah and Sarawak are considered biodiversity hotspots with large forest areas. Contact with infected arthropod vectors, including ticks, fleas, lice, and mites, may have contributed to the higher number of positive cases. A study conducted in 2000 in Nabawan, Sabah [26], reported 91.7% (n = 145) rickettsial seropositivity. Among these seropositive patients, 84.8% were positive for ST and SFGR (TT118), and 54.5% for TGR (*R. typhi*). In the same study, seropositivity was 66.1% (n = 322) in another locality in Selangau, a district in Sibu, Sarawak, where seropositivity for SFGR (TT118) was 59.6%. The two localities were rural and close to forests, oil palm, and rubber plantations. A recent study reported rickettsioses as major etiologies of acute febrile illness with confirmed cases of ST, TGR, and SFGR in Sabah [6]. The same study described that infections were more commonly found in cases with a history of forest exposure. A case study from Sabah, reported in 2018, described a patient infected with SFGR, and this infection was also associated with ecological and entomological factors. The patient had performed ecological research for 20 days in the forests of the Sandakan Division in Sabah, had multiple insect bites, reported being in close proximity to cats and dogs, and reported a large number of ticks a week prior to the onset of the fever [27].

The time series analysis of rainfall correlation indicates a potential association between higher rainfall and subsequent increases in rickettsial cases in Sarawak, particularly with a one-month lag. This pattern was most notable during periods following the main rainy season, consistent with previous observations that heavy rainfall events may increase vector activity and human exposure risk Previous studies in Teluk Intan also showed a correlation between rainfall and the surge in rickettsial cases (Yuhana et al., 2022) [28]. Employing finer-resolution data, such as weekly rainfall and case numbers, could provide a more accurate understanding of this temporal association. Further studies are warranted to confirm this relationship and to explore underlying ecological mechanisms, including vector abundance and human behavioral factors during and after periods of heavy rainfall.

In our IgM antibody analyses, females showed significantly higher seropositivity than males for SFGR. In contrast, the IgG seropositivity for SFGR of males was higher than that of females. However, a study of outdoor recreationists’ exposure to SFG rickettsia in Western Australia found no significant difference between genders [29]. Higher SFG rickettsiae cases among females were also observed in India [30]. The close proximity of pets and stray dogs to humans, where women come into contact with infected ticks, was the reason for the higher seropositivity observed among females [30]. Because the participating hospitals gave no clinical histories, we were unable to identify the reasons for the higher seropositivity among the females in this study. Although gender was insignificant in IgM antibodies for ST and TGR in our study, a higher incidence of ST cases among females was observed in Nepal [31], China [32], and Korea [33]. These studies reported that women tend to be at higher risk due to active involvement in agricultural activities, household chores, and tending to their gardens. Our findings showed that males had significantly higher IgG seropositivity for SFGR than females. Without clinical histories, we were unable to interpret the predominance of IgG seropositivity for SFGR in males compared to females. 

A significant inverse association was observed between IgM seropositivity for SFGR and age group, with significantly lower seropositivity observed in older age groups. The age group >65 years showed a substantially decreased IgM seropositivity compared to younger age groups. In contrast, IgG seropositivity for SFGR was significantly associated with increasing age, particularly in the 41–65-year and >65-year age groups. Without occupational data or clinical histories, we could not determine whether occupational factors or daily activities were associated with the seropositivity across age groups. 

Proper serological testing for the diagnosis of serological testing requires paired acute and convalescent sera collected 2–3 weeks apart to assess a 4-fold or greater rise in titer. Thus, paired IgG samples will confirm this region’s specific acute febrile illness rather than having IgM results as the confirmation test. Serological cross-reactions are often observed in IgM and may generate false-positive results. Therefore, collecting convalescent sera samples for serological testing would help increase the accuracy of diagnosis. With the availability of convalescent sera results, the treating physician can confirm rickettsioses in patients who presented late, that is, outside the bacteremia phase, and were unable to be tested by polymerase chain reaction (PCR) based test. Nevertheless, PCR is the first-line diagnostic test for cases presented within 7–10 days after the onset of illness. This molecular technique is a useful tool for accurate and rapid detection of rickettsioses, and can identify *O. tsutsugamushi* and *Rickettsia* spp. up to the species level. 

Our findings provide important contributions to the epidemiological characterization of potential endemic regions and high-risk populations for rickettsial diseases in Malaysia, thereby facilitating constructive differential diagnoses of acute febrile illness, particularly when other more common tropical diseases have been excluded. The serological data also provide insights into the distribution of prevalent *Rickettsia* species within specific regions and the demographic populations that are at higher risk. Such awareness can facilitate earlier diagnosis, which is critical for initiating timely and effective treatment to reduce the risk of severe complications and mortality. Moreover, these data can facilitate the implementation of targeted public health interventions by enabling the planning of prevention and control strategies, and guiding risk communication and educational campaigns led by public health authorities. This includes emphasizing practical measures such as wearing protective clothing and using insect repellents. 

The limitations of this study were the reliance on a single sample serum and the lack of complete clinical history, physical findings, or data on other tests performed, which hamper further analyses and interpretations of our findings. In addition, the seropositivity findings were based solely on clinically suspected cases submitted to public healthcare facilities. While the samples were obtained from all regions across Malaysia, the study population did not include asymptomatic individuals or those who did not seek medical attention. Hence, the results should not be generalized to the wider population.

Future research directions should include integrated vector surveillance and analysis of environmental determinants to better understand and manage rickettsial epidemiology. Serological evidence of human infection can serve as a trigger to intensify vector surveillance activities aimed at identifying specific tick, flea, or mite species involved in local transmission. This approach should incorporate both morphological identification and molecular testing of vectors to detect the presence of *Rickettsia* spp. Additionally, the collection and analysis of environmental data, such as rainfall, temperature, and humidity, can help to elucidate ecological factors that promote vector abundance and pathogen transmission. By integrating these data into spatial analyses, including the use of geographic information system (GIS) tools, high-risk areas can be more accurately mapped, thereby guiding targeted vector control strategies and optimizing the allocation of public health resources. 

## 5. Conclusions

In conclusion, this six-year analysis of ST, TGR, and SFGR seropositivity in Malaysia highlights notable regional and demographic disparities. Our findings contribute valuable insights into the largely underreported burden of rickettsial infections in Malaysia and reinforce the urgent need for enhanced diagnostic capacity, active surveillance systems, and community-based prevention strategies. By addressing the identified regional and demographic risk patterns, public health authorities can develop more tailored and effective interventions to control and ultimately reduce the impact of these re-emerging but neglected infections on affected populations.

## Figures and Tables

**Table 1:** Demographic characteristics of 3228 patients suspected of rickettsioses tested for seropositivity for ST, TG, and SFG (IgM and IgG), 2016–2021.

Characteristics		n	%
**Year**			
	2016	380	11.8
	2017	534	16.5
	2018	853	26.4
	2019	1002	31
	2020	165	5.1
	2021	294	9.1
Region			
	Peninsular Malaysia		
	Southern	176	5.5
	Johor	148	84.1
	Melaka	23	13.1
	Negeri Sembilan	5	2.8
	Northern	860	26.6
	Kedah	4	0.5
	Perak	843	98
	Penang	9	1
	Perlis	4	0.5
	Central	1107	34.3
	Kuala Lumpur	591	53.4
	Selangor	516	46.6
	East Coast	429	13.3
	Kelantan	172	40.1
	Pahang	13	3
	Terengganu	244	56.9
	East Malaysia		
	Sabah	279	8.6
	Sarawak	377	11.7
Gender			
	Female	1292	40
	Male	1936	60
Age group			
	<15	344	10.7
	15–40	1467	45.4
	41–65	1120	34.7
	>65	297	9.2

**Table 2:** Seropositivity for ST, TGR, and SFGR (IgM), 2016–2021.

Year	ST		TGR		SFGR	
	Positive	Negative	Positive	Negative	Positive	Negative
	n (%)	n (%)	n (%)	n (%)	n (%)	n (%)
2016	73 (19.2)	307 (80.8)	82 (21.6)	298 (78.4)	42 (11.1)	338 (88.9)
2017	75 (14.0)	459 (86.0)	117 (21.9)	417 (78.1)	100 (18.7)	434 (81.3)
2018	199 (23.3)	654 (76.7)	188 (22.0)	665 (78.0)	129 (15.1)	724 (84.9)
2019	300 (29.9)	702 (70.1)	96 (9.6)	906 (90.4)	166 (16.6)	836 (83.4)
2020	22 (13.3)	143 (86.7)	21 (12.7)	144 (87.3)	9 (5.5)	156 (94.5)
2021	27 (9.2)	267 (90.8)	15 (5.1)	279 (94.9)	4 (1.4)	290 (98.6)
Linear trend test	0.03		73.46		18.46	
*p*-value	0.861		<0.001		<0.001	

**Table 3:** Seropositivity for ST, TGR, and SFGR (IgG), 2016–2021.

Year	ST		TGR		SFGR	
	Positive	Negative	Positive	Negative	Positive	Negative
	n (%)	n (%)	n (%)	n (%)	n (%)	n (%)
2016	85 (22.4)	295 (77.6)	121 (31.8)	259 (68.2)	55 (14.5)	325 (85.5)
2017	80 (15.0)	454 (85.0)	195 (36.5)	339 (63.5)	126 (23.6)	408 (76.4)
2018	186 (21.8)	667 (78.2)	212 (24.9)	641 (75.1)	138 (16.2)	715 (83.8)
2019	285 (28.4)	717 (71.6)	115 (11.5)	887 (88.5)	207 (20.7)	795 (79.3)
2020	25 (15.2)	140 (84.8)	27 (16.4)	138 (83.6)	17 (10.3)	148 (89.7)
2021	45 (15.3)	249 (84.7)	20 (6.8)	274 (93.2)	13 (4.4)	281 (95.6)
Linear trend test	0.34		159.68		15.06	
*p*-value	0.561		<0.001		<0.001	

**Table 4:** Logistic regression analysis of demographic factors for ST seropositivity (IgM).

Demographic Factor	Univariate		Multivariable	
	COR (95%CI)	*p*-Value	AOR (95%CI)	*p*-Value
Region				
Southern	1		1	
Northern	4.05 (2.02, 8.09)	<0.001	4.05 (2.02, 8.10)	<0.001
Central	5.03 (2.53, 9.98)	<0.001	5.00 (2.52, 9.94)	<0.001
East Coast	3.56 (1.73, 7.30)	0.001	3.57 (1.74, 7.34)	0.001
Sabah	8.69 (4.25, 17.79)	<0.001	8.82 (4.31, 18.06)	<0.001
Sarawak	10.84 (5.37, 21.88)	<0.001	11.24 (5.56, 22.71)	<0.001
Gender				
Female	1		1	
Male	0.90 (0.76, 1.07)	0.241	0.84 (0.70, 1.00)	0.050
Age group				
<15	1		1	
15–40	0.95 (0.72, 1.26)	0.724	0.94 (0.70, 1.25)	0.650
41–65	0.89 (0.66, 1.18)	0.412	0.82 (0.61, 1.11)	0.206
>65	0.83 (0.57, 1.22)	0.341	0.84 (0.57, 1.24)	0.377

Hosmer and Lemeshow (*p* = 0.751), Classification Table (78.4%), Nagelkerke R Square (5.7%), and VIF < 5.0.

**Table 5:** Logistic regression analysis of demographic factors for TGR seropositivity (IgM).

Demographic Factor	Univariate		Multivariable	
	COR (95%CI)	*p*-Value	AOR (95%CI)	*p*-Value
Region				
Southern	1		1	
Northern	1.93 (1.08, 3.44)	0.026	2.03 (1.13, 3.62)	0.017
Central	2.32 (1.32, 4.10)	0.004	2.31 (1.31, 4.09)	0.004
East Coast	0.30 (0.14, 0.68)	0.004	0.30 (0.14, 0.69)	0.004
Sabah	4.49 (2.45, 8.23)	<0.001	4.52 (2.47, 8.29)	<0.001
Sarawak	4.65 (2.58, 8.38)	<0.001	4.72 (2.61, 8.52)	<0.001
Gender				
Female	1		1	
Male	1.01 (0.83, 1.22)	0.943	0.97 (0.79, 1.18)	0.757
Age group				
<15	1		1	
15–40	1.11 (0.80, 1.54)	0.521	1.03 (0.74, 1.44)	0.866
41–65	1.12 (0.81, 1.56)	0.497	0.97 (0.68, 1.37)	0.849
>65	0.59 (0.37, 0.96)	0.034	0.54 (0.33, 0.89)	0.016

Hosmer and Lemeshow (*p* = 0.864), Classification Table (83.9%), Nagelkerke R Square (8.8%), and VIF < 5.0.

**Table 6:** Logistic regression analysis of demographic factors for SFGR seropositivity (IgM).

Demographic Factor	Univariate		Multivariable	
	COR (95%CI)	*p*-Value	AOR (95%CI)	*p*-Value
Region				
Southern	1		1	
Northern	4.87 (1.51, 15.67)	0.008	5.17 (1.60, 16.64)	0.006
Central	10.61 (3.35, 33.60)	<0.001	10.38 (3.27, 32.89)	<0.001
East Coast	4.65 (1.40, 15.38)	0.012	4.60 (1.39, 15.25)	0.013
Sabah	21.2 (6.57, 68.42)	<0.001	21.93 (6.79, 70.84)	<0.001
Sarawak	21.1 (6.59, 67.58)	<0.001	22.53 (7.03, 72.25)	<0.001
Gender				
Female	1		1	
Male	0.88 (0.72, 1.08)	0.218	0.78 (0.63, 0.96)	0.019
Age group				
<15	1		1	
15–40	0.65 (0.48, 0.89)	0.006	0.65 (0.48, 0.89)	0.008
41–65	0.63 (0.46, 0.86)	0.004	0.60 (0.43, 0.83)	0.002
>65	0.30 (0.18, 0.51)	<0.001	0.33 (0.19, 0.56)	<0.001

Hosmer and Lemeshow (*p* = 0.754), Classification Table (86.1%), Nagelkerke R Square (10.0%), and VIF < 5.0.

**Table 7:** Logistic regression analysis of demographic factors for ST seropositivity (IgG).

Demographic Factor	Univariate		Multivariable	
	COR (95%CI)	*p*-Value	AOR (95%CI)	*p*-Value
Region				
Southern	1		1	
Northern	6.08 (2.80, 13.19)	<0.001	5.91 (2.72, 12.84)	<0.001
Central	5.32 (2.46, 11.51)	<0.001	5.44 (2.51, 11.76)	<0.001
East Coast	6.05 (2.74, 13.37)	<0.001	6.19 (2.80, 13.69)	<0.001
Sabah	10.76 (4.85, 23.88)	<0.001	10.86 (4.89, 24.12)	<0.001
Sarawak	16.67 (7.62, 36.49)	<0.001	16.19 (7.39, 35.47)	<0.001
Gender				
Female	1		1	
Male	1.08 (0.91, 1.29)	0.358	1.02 (0.85, 1.22)	0.827
Age group				
<15	1		1	
15–40	1.12 (0.82, 1.51)	0.479	1.06 (0.78, 1.45)	0.710
41–65	1.55 (1.14, 2.10)	0.005	1.39 (1.02, 1.91)	0.039
>65	1.43 (0.97, 2.10)	0.068	1.38 (0.93, 2.05)	0.114

Hosmer and Lemeshow (*p* = 0.206), Classification Table (78.1%), Nagelkerke R Square (6.9%), and VIF < 5.0.

**Table 8:** Logistic regression analysis of demographic factors for SFGR seropositivity (IgG).

Sociodemographic Factor	Univariate		Multivariable	
	COR (95%CI)	*p*-Value	AOR (95%CI)	*p*-Value
Region				
Southern	1		1	
Northern	2.52 (1.25, 5.09)	0.010	2.46 (1.22, 4.97)	0.012
Central	3.37 (1.69, 6.71)	0.001	3.44 (1.72, 6.87)	0.000
East Coast	1.50 (0.70, 3.20)	0.300	1.50 (0.70, 3.21)	0.300
Sabah	9.89 (4.84, 20.21)	<0.001	9.85 (4.81, 20.17)	<0.001
Sarawak	11.60 (5.75, 23.40)	<0.001	10.87 (5.38, 21.97)	<0.001
Gender				
Female	1		1	
Male	1.46 (1.2, 1.77)	<0.001	1.36 (1.11, 1.66)	0.003
Age group				
<15	1		1	
15–40	1.26 (0.89, 1.80)	0.197	1.18 (0.82, 1.70)	0.378
41–65	1.95 (1.37, 2.78)	<0.001	1.75 (1.21, 2.53)	0.003
>65	1.67 (1.08, 2.58)	0.021	1.77 (1.12, 2.80)	0.014

Hosmer and Lemeshow (*p* = 0.582), Classification Table (82.8%), Nagelkerke R Square (12.5%), and VIF < 5.0.

**Table 9:** Logistic regression analysis of demographic factors for TGR seropositivity (IgG).

Demographic Factor	Univariate		Multivariable	
	COR (95%CI)	*p*-Value	AOR (95%CI)	*p*-Value
Region				
Southern	1		1	
Northern	2.56 (1.53, 4.27)	<0.001	2.53 (1.51, 4.23)	<0.001
Central	2.13 (1.28, 3.54)	0.004	2.17 (1.30, 3.62)	0.003
East Coast	0.57 (0.30, 1.06)	0.076	0.57 (0.31, 1.08)	0.084
Sabah	5.30 (3.07, 9.13)	<0.001	5.30 (3.07, 9.14)	<0.001
Sarawak	4.67 (2.75, 7.95)	<0.001	4.46 (2.62, 7.61)	<0.001
Gender				
Female	1		1	
Male	1.14 (0.96, 1.36)	0.133	1.15 (0.96, 1.37)	0.138
Age group				
<15	1		1	
15–40	1.38 (1.00, 1.91)	0.048	1.25 (0.90, 1.74)	0.190
41–65	1.90 (1.37, 2.63)	<0.001	1.62 (1.16, 2.27)	0.005
>65	1.57 (1.05, 2.35)	0.028	1.39 (0.91, 2.11)	0.126

Hosmer and Lemeshow (*p* = 0.841), Classification Table (78.6%), Nagelkerke R Square (8.6%), and VIF < 5.0.

## Data Availability

The research data for this study is presented in Supplementary Materials S1 and S2 Document Dataset.

## Supplementary Materials

The following supporting information can be downloaded at https://www.mdpi.com/article/10.3390/tropicalmed10080205/s1, Supplementary S1: Document Dataset (CSV); Supplementary S2: Document Dataset (pdf).
