# Supplementary material for: Rickettsioses Seropositivity in Malaysia: A Six-Year Trend, 2016–2021
Source: Trop Med Infect Dis. 2025 Jul 24;10(8):205. doi: 10.3390/tropicalmed10080205 (PMC12390281; doi:10.3390/tropicalmed10080205)
Supplement: Supplementary file 1 [file tropicalmed-10-00205-s001.zip › S2 Document Dataset.pdf]

Dataset Title: Monthly Rainfall Amount and Rickettsioses Cases in Kuching, Sarawak, Malaysia from June 2016 to December 2019

Data Description:

This dataset includes two components

1. Monthly Rickettsioses Case Counts, and
2. 90th Percentile Monthly Rainfall Amounts, aggregated from daily rainfall data.

Rainfall data were collected by the Malaysian Meteorological Department from 08:00 to 08:00 MST daily in Kuching, Sarawak, Malaysia. The 90th percentile values represent monthly summaries of extreme rainfall events, calculated from daily measurements between June 2016 and December 2019. The rainfall data were obtained from the Malaysian Meteorological Department upon formal request.

Rickettsioses case counts for the same period in Sarawak were sourced from The Institute for Medical Research, providing a basis for examining potential environmental influences on disease incidence.

Column Descriptions:

1. Month – MM-YY format
2. P90_rainfall – 90th percentile monthly rainfall amount in mm
3. Rickettsioses_cases – count of confirmed ST, TGR, and SFGR cases

Contact Information:

Norli Anida Abdullah (norlie@um.edu.my)

Month	Rickettsioses_cases	P90_rainfall
Jun-16	4	34.60
Jul-16	2	33.30
Aug-16	3	33.30
Sep-16	4	31.26
Oct-16	10	33.30
Nov-16	6	34.47
Dec-16	6	21.60
Jan-17	4	43.40
Feb-17	1	47.90
Mar-17	7	38.00
Apr-17	2	43.86
May-17	4	33.30
Jun-17	6	33.30
Jul-17	4	31.60
Aug-17	1	33.30
Sep-17	5	33.30
Oct-17	5	33.30
Nov-17	5	33.30
Dec-17	2	33.30
Jan-18	5	33.30
Feb-18	1	40.32
Mar-18	5	33.30
Apr-18	10	34.71
May-18	2	21.40
Jun-18	8	33.43
Jul-18	3	36.40
Aug-18	4	25.80
Sep-18	1	33.30
Oct-18	3	33.30
Nov-18	2	33.61
Dec-18	5	47.20
Jan-19	11	42.20
Feb-19	7	33.30
Mar-19	9	23.60
Apr-19	9	33.30
May-19	8	33.30
Jun-19	9	33.51
Jul-19	9	33.30
Aug-19	6	24.00
Sep-19	7	27.81
Oct-19	5	33.30
Nov-19	8	33.30
Dec-19	4	56.20
